# Vulnerability to extreme heat and climate change: is ethnicity a factor?

**DOI:** 10.3402/gha.v6i0.21364

**Published:** 2013-07-29

**Authors:** Alana Hansen, Linda Bi, Arthur Saniotis, Monika Nitschke

**Affiliations:** 1 Discipline of Public Health, University of Adelaide, Adelaide, Australia; 2 The South Australian Department for Health and Ageing, South Australia

**Keywords:** Australia, barriers, climate change, ethnicity, heat-susceptibility, migrants

## Abstract

**Background:**

With a warming climate, it is important to identify sub-populations at risk of harm during extreme heat. Several international studies have reported that individuals from ethnic minorities are at increased risk of heat-related illness, for reasons that are not often discussed.

**Objective:**

The aim of this article is to investigate the underpinning reasons as to why ethnicity may be associated with susceptibility to extreme heat, and how this may be relevant to Australia’s population.

**Design:**

Drawing upon literary sources, the authors provide commentary on this important, yet poorly understood area of heat research.

**Results:**

Social and economic disparities, living conditions, language barriers, and occupational exposure are among the many factors contributing to heat-susceptibility among minority ethnic groups in the United States. However, there is a knowledge gap about socio-cultural influences on vulnerability in other countries.

**Conclusion:**

More research needs to be undertaken to determine the effects of heat on tourists, migrants, and refugees who are confronted with a different climatic environment. Thorough epidemiological investigations of the association between ethnicity and heat-related health outcomes are required, and this could be assisted with better reporting of nationality data in health statistics. Climate change adaptation strategies in Australia and elsewhere need to be ethnically inclusive and cognisant of an upward trend in the proportion of the population who are migrants and refugees.

High-temperature days can pose a threat to human health by either triggering acute heat-related illnesses or by exacerbating pre-existing health conditions (1). However, adverse health outcomes can often be prevented by simple adaptive behaviour. The vulnerability of communities to extreme heat can be influenced by social characteristics and population composition, but these factors can often be neglected when guidelines and policies are formulated (2). With future scenarios predicting a climate-change-driven increase in the frequency and intensity of heatwaves, there is an increased need for identification of vulnerable sub-groups and evidence-based adaptation and prevention strategies to boost resilience in those at risk (3).

Whilst it is well established that amongst the most at risk during hot weather are the aged and the very young, some studies from North America have identified increased vulnerability in ethnic minority groups (2, 4). This may become a climate change issue of public health importance in countries such as Australia with its warming climate and increasing level of cultural diversity. Two recent studies from Adelaide, South Australia, have indicated a potential vulnerability to heat in people born outside Australia. One noted that older people of migrant backgrounds could be more at risk (5), and another reported that 37% of patients hospitalised with direct heat-related illnesses during an extreme heatwave were non-Australian born (6), despite only 25% of the city’s population being born overseas (7). Furthermore, a recently released Australian report investigating spatial vulnerability indicated that ethnicity featured strongly as a risk factor for heat-related adverse health outcomes in some Australian cities (8).

Overall, few studies have specifically addressed the issue of ethnicity as a risk factor for heat-related mortality and morbidity (2), indicating a knowledge gap which needs to be addressed in the face of climate change. The aim of this article is to investigate the underpinning reasons as to why ethnicity may be associated with susceptibility to extreme heat, and how this may be relevant to Australia’s population. Literary evidence was sourced from relevant peer-reviewed and grey literature.

## Risk factors for ethnic minority groups during heatwaves

Over recent decades, there has been increased interest in the scientific literature about sub-populations susceptible to extreme heat. Certain ‘non-White’ minority groups in North America are among those with reportedly higher morbidity and mortality rates associated with hot weather (2, 4). Heatwave studies in the United States, for instance, have shown that African Americans are particularly vulnerable, with the mortality rate during a Los Angeles heatwave being nearly double that of the city’s average (9). Similar findings have emerged from heatwave studies in other US cities where the risk of mortality has been demonstrated to be higher for African American residents (10–16), but not necessarily for people of Hispanic backgrounds. During the 1995 heatwave in Chicago during which 739 excess deaths were recorded, age-adjusted death rates showed that African Americans were disproportionally represented in the heat statistics and were 1.5 times more likely to die than Whites, and almost 30 times more likely than Latinos (11, 13, 17). One suburb with a high mortality rate was composed of mainly (96%) African American residents, whereas in another where 85% of the residents were Latino, the mortality rate was much lower (13). The nature of the suburbs had a large bearing on the health impacts. The higher death rate occurred in an abandoned, dangerous neighbourhood with a history of violent crime such that people were afraid to leave their homes – unlike in the adjacent suburb with its high social capital amongst Latino families and busy commercial activity (17).

However, findings vary between studies. In an investigation of hospitalisations and deaths from heat illness in US soldiers, it was found that African Americans and Hispanic Americans were less likely than Caucasians to be hospitalised for heat-related illness (18). During an extreme heatwave in California in 2006, increases in the rate of emergency department (ED) visits were found for most ethnic groups, including people in the African American and Latino/Hispanic groups, as well as Whites (19). Heat-related ED visits increased for Asian/Pacific Islanders and African Americans, and for the latter there were also significant increases for conditions including acute renal failure, electrolyte imbalance and nephritis (19).

There have been very few similar studies outside the United States. Although numerous reports emerged following the 2003 heatwave in Europe, the collected data did not apparently include ethnicity or cultural background. Capturing this information about heat casualties is important to determine the extent to which ethnicity and population characteristics contribute to vulnerability during heatwaves.

### Ethnicity

Classifying individuals or communities according to race/ethnicity for epidemiological research is problematic on many levels. Race generally refers to certain phenotypic traits such as skin colour (20), whilst ethnicity is a broader term defined as ‘the shared identity or similarity of a group of people on the basis of one or more factors’, and can relate to shared history or cultural tradition, country of origin, common language, literature, minority group or being ‘racially conspicuous’ (21). However, as race/ethnicity is part of an individual’s identity, it can be subjective and ideally should be defined by self-report (20).

The link between racial/ethnic status and the likelihood of poor heat health outcomes is multidimensional. Despite apparent disparities in heat-related morbidity and mortality rates, there is no evidence to suggest that any difference in heat loss responses between races would confer physiological advantage during extreme heat (2). Although some differences have been noted between the number of sweat glands in people of different ethnicities, little or no difference has been found between Caucasians and African American individuals in terms of sweat loss under the same experimental conditions (22). Instead, other factors including genetic adaptation and acclimatisation could play a role in differences between cultural groups (23). In addition, health inequities in minority groups can also be exacerbated by race and class discrimination, as well as social problems (24). The role of modifiable rather than non-modifiable risk factors in the disparities between heat-associated health outcome rates in different ethnicities therefore deserves further investigation.

### Socio-economic issues

Major contributors to health inequalities are the social determinants of health, including the social and economic conditions that affect people’s lives, educational attainment, and the environment in which they live and work (25). Minority ethnic groups in the United States are often less educated, more likely to be socio-economically disadvantaged and work in higher risk occupations, and more likely to live in hazardous areas (26). With clear causal pathways linking race, socio-economic status (SES), and poor health outcomes (20), differences in vulnerability may be attributed to social and economic disparities rather than, or as well as, ethnicity (24, 27).

Persons with relative social disadvantage can be disproportionately affected by temperature extremes (16), particularly if there are financial impediments to maintaining thermal comfort. In a study comprising 50 US cities, it was noted that low SES overlapped with susceptibility to extreme heat in African Americans and poorly educated people (28). During the 1980 heatwave in St. Louis and Kansas City, higher rates of heat stroke were observed in non-Whites. However, low SES was a potential confounder as heat stroke rates were considerably higher in the lowest socio-economic areas (29). Socio-economic vulnerability has also been found to be related to the risk of heat deaths and heat distress calls elsewhere (30, 31). One study found there were more heat distress calls made from neighbourhoods with a higher proportion of African American residents, as well as low-income Hispanic residents who were often linguistically isolated and living in rental accommodation (30). Low SES, poor housing conditions, and overcrowding are likely to be contributing factors to the higher proportion of heat-related deaths in these groups (12).

Disparities in spatial vulnerability have been described. The financially disadvantaged, the less educated, and ethnic minorities are reportedly more likely to live in warmer neighbourhoods (32, 33), despite often lacking the social and material resources to cope with high temperatures (34). This notion was supported by a study of the urban heat island effect during an intense heatwave in Oklahoma City. It was found that in the warmer inner city region, the older housing stock was not adequately air-conditioned and was populated by many who were disadvantaged, including minority racial groups (35).

Using an air-conditioner is protective against the risk of heat-illness (36, 37). In the United States, an inverse relationship has been found between air-conditioning ownership and mortality, with excess heat-related mortality ‘reducing by 1.14 deaths per year (per standard million) for every percentage increase in home air conditioning availability’ (38). Indeed, over time the increased prevalence of air conditioners (2) has contributed to a decline in heat-related mortality rates in the US (38). However, the increasing cost of electricity required to run air-conditioners can be a barrier for the socio-economically disadvantaged (2). With the prevalence of central air-conditioning among African American households being less than half that of White households in some US cities (36), it follows that the disparities in the incidence of heat-related deaths by race could be partially attributed to differences in access to air-conditioning (39).

### Occupational factors

People in certain occupations are at higher risk during times of extreme heat, particularly those required to work outdoors (27). Heat stress can be a major occupational hazard in the US agricultural industry, for example, where language and cultural barriers have an impact on workplace safety. The agricultural workforce consists of people from diverse ethnic backgrounds, with the majority being from Latin America. There is also a significant proportion who are of African American and Native American descent, as well as Asian and Caribbean migrants (40).

An investigation of a 2006 Californian heatwave found significantly elevated rates of hospitalisation and ED rates for cardiac-related illness in people from Latino/Hispanic backgrounds, and the authors postulated that this could be due to occupational heat exposures in crop workers (19). A coordinated effort is required to develop culturally, linguistically, and literacy appropriate occupational health and safety training programmes for migrant workers in the agricultural industry (40).

### Language barriers

Members of ethnic minority language groups can be doubly vulnerable through poor living conditions and exclusion from access to English-based media and health messages (41). The linguistic barrier faced by those who are not proficient in the main language of the country can affect their ability to follow weather reports and instructions from government organisations and service providers, including information aimed to increase awareness and reduce the impact of excessive heat (15). Accordingly, this can hinder understanding of heat warnings, potentially leading to reduced uptake of adaptive behaviour messages and greater risk of heat distress (30).

## Relevance to Australia

The international literature on the association between heat-susceptibility and ethnicity may not be totally applicable to the Australian context. Whilst terms such as ethnicity, race, and migrancy are often used interchangeably, meanings can differ between populations. For example, in the United States the term ‘migrant’ often refers to people from Mexico or Central America. Some who enter the United States illegally do so by crossing the United States–Mexico border in Arizona and many suffer the fatal effects of heat exposure when crossing the Arizona desert (42, 43). In Australia, the term ‘migrant’, or ‘immigrant’ (i.e. someone intending to be a permanent resident) has different connotations and generally refers to people or their descendants, who were born in countries ‘overseas’.

People of Aboriginal and/or Torres Strait Islander origin constitute 2.5% of Australia’s population (44). A recent study has shown temperature sensitivity in Indigenous Australians living in a remote community where ‘hot temperatures substantially increased the risk of hospitalization’ (45). Inadequate housing and facilities, culturally inappropriate medical services, and high prevalence of chronic diseases are some of the many factors which can increase the risk of harm for Indigenous Australians during hot spells, particularly for those living in remote regions (46).

Australia’s long history of immigration has contributed to demographic change such that the population is now highly culturally and linguistically diverse (CALD). Australian Bureau of Statistics figures show that in 2011, 27% of the estimated residential population of Australia was born overseas, compared with 23.1% ten years earlier (47). In the early post-war years, the majority of migrants were from the British Isles and Europe (48), and in the 1960s and 1970s many arrived from South-East Asia. In the 1990s, there was a higher proportion of migrant arrivals from the former Soviet Union, Eastern Europe, the Middle East, and Asia (48). In the first decade of the 20th century there was a high migrant intake from Asia, Nepal, India, Sudan, Zimbabwe, Bangladesh, and Pakistan (49), many of whom were refugees from their home countries. The number of people seeking protection in Australia increased from approximately 2,000 in the period 1976–1981, to almost 12,200 between 1999 and 2001 (50). Most (76%) of the recent migrants originate from non-English-speaking countries (50). The 2011 national census showed that 49% of longer-standing migrants and 67% of recent arrivals reported speaking a language other than English at home, with 3.1% of recent arrivals speaking no English at all (51).

### Potential barriers for people born overseas during hot weather in Australia

Whether the reported socio-cultural barriers contributing to racial/ethnic disparities in heat susceptibility in the United States are applicable to Australia remains to be explored. For groups such as humanitarian entrants, linguistic barriers, socio-economic disadvantage, and unemployment could potentially modify risks arising during hot weather. However, for people who enter the country under programmes to encourage skilled migrants, employment rates are comparable to those of other Australians (50). Notwithstanding, immigrants generally have incomes that commence lower than those of people born in Australia (48), and compared to those from English-speaking countries, unemployment rates for recent migrants are higher for those from non-English-speaking countries (50). With unemployment slowing the acquisition and improvement of English proficiency skills (48), 33% of recent migrants have reported language barriers when trying to gain employment (50). This lack of financial resources contributes to lower SES and may influence susceptibility to extreme heat as outlined above. Financial handicap was shown to be a barrier to accessing health care in a study of newly settled refugees in Sydney. Others included language barriers, not knowing how and where to get help and access health services, and lack of health information (52). It is not only the newly arrived who may lack English proficiency skills, as language barriers can be an issue for Australia’s post-war migrants from non-English-speaking countries. Whilst individuals may be proficient in a second language during their younger years, reversion to their first language can occur later in life (53), particularly for those with cognitive decline. This can contribute to linguistic isolation and can increase susceptibility to extreme heat in older migrants (5).

Furthermore, new arrivals and tourists, particularly those from cooler countries, may be unacclimatised to high temperatures and lack awareness of adaptive behaviours. Indeed, an examination of a series of heat-related deaths in Australia noted that four of the nine were tourists, and that being unfamiliar with the harsh climate was a contributing factor in their deaths (54). Despite the nation’s hot summers, there is a paucity of literature regarding vulnerability to heat in foreign-born people in Australia. This may be partly due to the fact that migrants lacking English proficiency skills can be under-represented in health research (55).

## Adaptation strategies for climate change

Whereas projections have stated that due to global warming, there will be an increase in the frequency, intensity, and duration of extreme heat events (56), a recent study of decadal trends has shown this to be occurring already (57). In Australia, temperatures are projected to increase by up to 1.5°C by 2030 (58). Climate change will impact on the social determinants of health and exacerbate health disparities (59). Those in poor health who are financially disadvantaged and already at risk of heat-related morbidity and mortality will face extra difficulties in a warmer climate. Climate change is expected to place a disproportionate burden on the physical and psychological health of Indigenous Australians (46), who have lived with historically based disadvantage since colonisation. Despite acclimatisation to hot conditions, vulnerability to climate change will likely be intensified by socio-economic factors and health disparities (44, 46).

The projected impact of climate change on the health of migrants and visitors to Australia is yet to be reported. However, in a Canadian study using Geographical Information Systems to locate groups susceptible to climate change and heatwaves, low-income immigrants were among the main groups identified (60). The study suggested that short-term adaptation strategies for climate change should include an initial assessment of the community to identify those at risk, increasing air-conditioning in public spaces for those with low income to access these resources, disaster response plans, and outreach to immigrant populations about heat warning systems and programmes (60).

Culturally and socially appropriate public service announcements about dangers and preventive strategies of heat stress to people in ethnic minorities can help save lives and reduce morbidity (19). Following major heatwaves in the recent years, heatwave action plans similar to those formulated by the World Health Organisation (27) are now common in countries around the world, and it has been recognised that early warning messages should be targeted at vulnerable groups including ethnic minorities and tourists (61). For example, the Contingency Plan for Excessive Heat Emergencies in California outlines recommendations for identifying vulnerable populations, developing strategies for notification, and increasing awareness by establishing processes to quickly disseminate extreme heat emergency advice to vulnerable populations including immigrant groups (62). Some Australian heatwave plans have also identified increased risk of heat-related illness in those who are not acclimatised, those with low SES, and non-English-speaking people (63, 64). A multimedia approach incorporating radio, television, and newspapers is most effective to ensure that a public warning system reaches the largest audience. For many recent migrants who may not have access to the broadcast media, networks of family and friends, as well as social workers and charity groups can assist in information dissemination (41).

In conclusion, past and present studies have noted that ethnicity and/or race may modify the risk of heat-related illness. Low SES, language barriers, occupational exposure, and spatial vulnerability are reported to be contributing factors, more so than ethnicity itself. However, more thorough epidemiological investigations of the association between ethnicity and heat-related health outcomes are required, and better reporting of nationality and country of birth in health statistics data would assist in this endeavour. In Australia (and elsewhere), climate change adaptation strategies need to be ethnically inclusive in the face of a projected increase in the number of hot days (58) and an upward trend in the proportion of the population who were born overseas (47). This may be useful to develop strategies and policy decisions to ensure equity in access to preventive heat health information, and assist population-wide adaptation to a warmer climate.
